# Large infinities and definable sets

**DOI:** 10.1073/pnas.2528175123

**Published:** 2026-04-09

**Authors:** J. P. Aguilera, J. Bagaria, P. Lücke

**Affiliations:** ^a^Institut für diskrete Mathematik und Geometrie, Technische Universität Wien, Vienna 1040, Austria; ^b^Institució Catalana de Recerca i Estudis Avançats, Barcelona 08010, Spain; ^c^Departament de Matemàtiques i Informàtica, Universitat de Barcelona, Barcelona, Catalonia 08007 Spain; ^d^Fachbereich Mathematik, Universität Hamburg, Hamburg 20146, Germany

**Keywords:** mathematical logic, large cardinal axiom, set theory, Axiom of Choice, philosophy of mathematics

## Abstract

Large cardinal axioms extend the standard set of axioms for mathematics by asserting that very large infinite sets exist. A prominent line of current research in mathematical logic is identifying ever stronger principles of this kind; this serves as an avenue toward mitigating the phenomenon of Gödel incompleteness. However, there is a tension between large cardinal axioms and principles asserting global simplicity of the mathematical universe, as well as forms of the Axiom of Choice. New kinds of infinity recently identified shed light into this tension and raise important mathematical and philosophical questions.

According to Gödel’s incompleteness theorems, there can exist no complete, consistent, computable axiomatization for mathematics. In particular, the standard set of axioms for set theory, the Zermelo–Fraenkel axioms (ZFC, including the Axiom of Choice), is incomplete. This is exemplified by the well-known theorem of Gödel (1) and Cohen (2) whereby Cantor’s Continuum Hypothesis is independent of the ZFC axioms.

One of the central goals of contemporary set theory is the identification of compelling new axioms for mathematics which make it possible to settle otherwise undecidable problems in a satisfactory manner. “Large cardinal axioms” assert that very large sets exist and arguably reflect our intuitions about infinity in mathematics. These principles provide canonical extensions of ZFC which are larger and larger in Gödel’s consistency strength ordering. If T and U are computable theories (i.e., collections of axioms), we write $T{<}_{\mathrm{con}}U$ if the consistency of T can be proved by U. Gödel’s incompleteness theorem can be rephrased by saying that the ordering ${<}_{\mathrm{con}}$ is irreflexive and unbounded. As large cardinal axioms increase the consistency strength of ZFC, they cannot be proved to hold, and, moreover, they cannot be proved to be consistent (i.e., not resulting in contradictions). Nonetheless, they can be put to test by studying their consequences and behavior.

Large cardinal axioms allow us to prove the consistency of other kinds of axioms, many of which bear direct impact on other domains of mathematics, such as Martin’s Maximum [see Foreman–Magidor–Shelah (3)] or the Axiom of Determinacy [see Woodin (4)]. Other axioms impose conditions of global “regularity” or “simplicity” within the set-theoretic universe, V. These include Gödel’s axiom V=L (1) according to which every set is constructible, or his axiom V=HOD, according to which every set is definable from ordinal numbers. V=HOD can be regarded as a strong form of the Axiom of Choice, and V=L can in turn be regarded as a strong form of V=HOD. According to a theorem of Scott (5), the axiom V=L is incompatible with the existence of sufficiently large cardinals.

## Scott’s Inconsistency Theorem.

The theory ZFC + “there exists a measurable cardinal” +V=L is inconsistent.

Here we recall Ulam’s notion of a “measurable cardinal” as that of a cardinal κ for which there is a total two-valued, κ-additive measure on the powerset of κ. Equivalently, if there is an elementary (i.e., truth-preserving) embedding from the set-theoretic universe V to a transitive class M which has critical point κ, where the critical point of an embedding j is the least α such that j(α)≠α. The idea of Scott’s inconsistency theorem is that large cardinals by nature impose indescribability and repetition in the set-theoretic universe, and this is incompatible with the regularity of constructible sets. However, V=L is compatible with weaker large cardinal notions such as weakly compact cardinals, introduced by Erdős and Tarski. A cardinal κ is “weakly compact” if the partition relation $\kappa \to {\left(\kappa \right)}_{2}^{2}$ holds; equivalently, if κ is inaccessible and for every transitive M closed under <κ-sequences there is a transitive N closed under <κ-sequences and an elementary embedding j:M→N with critical point κ. Thus, weak compactness is a weakening of measurability.

According to our intuition on the notion of “set,” large infinities appear to be natural assumptions. This intuition is bolstered by their proven usefulness for mathematics. Thus, Scott’s inconsistency theorem is often interpreted as evidence against V=L as an axiom. Much current research in set theory is focused on the search for axioms which combine the advantages of large cardinals with those of V=L.

The hypothesis of measurable cardinals can be strengthened by demanding the existence of elementary embeddings from the set-theoretic universe V into classes M which resemble V more and more closely, with the intuition being that with the existence of large infinities, patterns in V repeat one way or another. The ulti- mate hypothesis in this direction is that of a Reinhardt cardinal: the critical point of an elementary embedding j:V→V. Kunen’s inconsistency theorem asserts that even a weak form of this principle is incompatible with the Axiom of Choice.

## Kunen’s Inconsistency Theorem.

The theory ZFC + “there is a nontrivial elementary embedding $j:V_{\lambda +2}\to V_{\lambda +2}$ for some λ” is inconsistent.

Here, $V_{\alpha }$ denotes the collection of all sets of rank <α (the “rank” of a set is defined inductively as the strict supremum of ranks of its elements). Kunen’s theorem did not rule out the hypothesis that nontrivial elementary embeddings $j:V_{\lambda }\to V_{\lambda }$ or $j:V_{\lambda +1}\to V_{\lambda +1}$ exist; these hypotheses are denoted I3 and I1, respectively. The principles I2 and I0 are strengthenings of I3 and I1, respectively, to which we will return later. Note that by Kunen’s theorem, if $j:V_{\lambda +1}\to V_{\lambda +1}$ is an I1 embedding, then necessarily λ must be a limit ordinal of countable cofinality and a fixpoint of j.

For decades, Kunen’s inconsistency theorem was interpreted as evidence for the fact that nontrivial elementary embeddings $j:V_{\lambda +2}\to V_{\lambda +2}$ and other principles incompatible with ZFC are also inconsistent with ZF (without the Axiom of Choice) and for the inconsistency of I3 and I1. Recently, opinion has begun to shift gradually, as these axioms have been studied more rigorously and their consequences tested, although the possibility remains (and will forever remain) that inconsistencies may arise in the future.

## Beyond the Axiom of Choice.

**Fig. 1.**
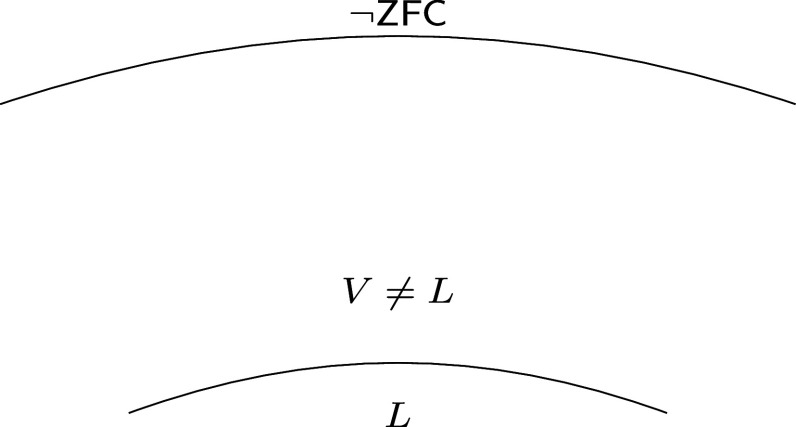
Former view of dividing lines in the large-cardinal hierarchy.

For decades, the landscape of infinity appeared to be depicted in Fig. 1: The weakest large cardinal hypotheses are compatible with V=L and the strongest are incompatible with the Axiom of Choice. This motivated the rigorous study of large cardinals beyond the Axiom of Choice [see e.g., Bagaria–Koellner–Woodin (6)]. One example is Schlutzenberg’s notion of a rank-Berkeley cardinal: λ is “rank-Berkeley” if for all α>λ there is an elementary embedding $j:V_{\alpha }\to V_{\alpha }$ with j(λ)=λ and critical point less than λ. As these principles are understood better, set-theorists become more confident in their consistency with ZF, but opinions are still divided.

The picture depicted by Fig. 1 has gradually changed, catalyzed by Schlutzenberg’s remarkable theorem (7) that the theory ZFC + “there is a nontrivial elementary embedding $j:V_{\lambda +2}\to V_{\lambda +2}$” is equiconsistent with the theory ZFC + I0. In other words, the large cardinal principles which are compatible with the Axiom of Choice overlap with those which are not.

## A Third Inconsistency Theorem

Reporting on recent work, we describe a large cardinal notion leading to a third, intermediate inconsistency theorem.

Definition 1. A cardinal λ is called “exacting” if for all α>λ there is an elementary substructure $X≺V_{\alpha }$ with $V_{\lambda }\cup \left\{\lambda \right\}\subset X$ and an elementary embedding $j:X\to V_{\alpha }$ with critical point $\mathrm{crit}\left(j\right)<\lambda$ and j(λ)=λ.

Exacting cardinals are obtained from rank-Berkeley cardinals by restricting the domain of the embedding, much like weakly compact cardinals are obtained from measurable cardinals. This situation is represented by the analogy

$$\begin{array}{c} \frac{\text{Exacting}}{\text{Rank-Berkeley}}\;=\;\frac{\text{Weakly compact}}{\text{Measurable}}. \end{array}$$

There are other pairs of large cardinal notions obeying this relation, e.g., strongly unfoldable and supercompact cardinals. The following theorem is proved in ref. (8, Theorem 2.10):

Theorem 1. *The theory* ZFC + *“there exists an exacting cardinal”* + V=HOD *is inconsistent.*

According to Theorem 1, there are large cardinal notions which directly imply the existence of undefinable sets. Compare this fact with the following theorem, proved in joint work with Goldberg ((9, Theorem E)) and strengthening a result of ((8, Theorem 2.9)):

Theorem 2. *The theory* ZFC + *I2 proves the consistency of* ZFC + *“there exists an exacting cardinal.” This theory in turn proves the consistency of* ZFC + *I3*.

The theorem in ref. 9 in fact places exacting cardinals at the bottom of the iterability hierarchy above I3 (see ref. 10). Thus, the region of large cardinals beyond HOD overlaps with that of those compatible with HOD, just like the region of large cardinals beyond the Axiom of Choice overlaps with that of those compatible with it. A strengthening of exacting cardinals called “ultraexacting cardinals” results from adding the condition $j↾V_{\lambda }\in X$ to the definition of exacting cardinals. Strengthening a result in ref. (8, Theorem 3.30), it is proved in joint work with Goldberg ((9, Theorem A)), that the existence of ultraexacting cardinals is equiconsistent with I0 (and thus with ZF+ the existence of an elementary embedding $j:V_{\lambda +2}\to V_{\lambda +2}$).

## The HOD Dichotomy and a Fourth Inconsistency

We now describe a fourth inconsistency theorem resulting from the combination of exacting cardinals with “old” large cardinals compatible with V=HOD. The background for this inconsistency theorem are the dichotomy theorems of Jensen and Woodin. Jensen’s theorem asserts that the universe V is either “very close” to L or “very far” from it, as reflected in its cardinal structure.

### The Jensen Dichotomy Theorem.

Precisely one of the following holds:

1. $\gamma^{+}=\gamma^{+L}$ for every singular cardinal γ, or
2. every uncountable cardinal is inaccessible in L.

Woodin’s HOD dichotomy theorem asserts a similar fact for HOD. Namely, that the universe V is either “very close” to HOD or “very far” from it. We state it as strengthened by Goldberg (11):

### The HOD Dichotomy Theorem.

Suppose that δ is a strongly compact cardinal. Precisely one of the following holds:

1. $\gamma^{+}=\gamma^{+HOD}$ for every singular cardinal γ>δ, or
2. every sufficiently large regular cardinal is measurable in HOD.

The first (“close”) alternative of the dichotomy is called the “HOD Hypothesis”. Woodin’s “HOD Conjecture” embodies the belief that the dichotomy is vacuous; it asserts that the HOD Hypothesis is provable from large cardinal axioms. Observe that the negation of the HOD Conjecture is a consistency assertion, and thus cannot be proved outright, by Gödel’s incompleteness theorem. However, opinions are divided on whether the HOD Conjecture itself is true (and provable) or false (but formally irrefutable). The fourth inconsistency theorem (8, Conclusion 6.8) could yield light into this issue:

Theorem 3. *The theory* ZFC + *The HOD Hypothesis* + *“there is an exacting cardinal above a strongly compact cardinal” is inconsistent.*

The following conclusion is drawn from Theorem 3: Either the HOD Conjecture is false or else the theory ZFC + “there is an exacting cardinal above a strongly compact cardinal” is inconsistent. Woodin (12, Theorem 200) had previously considered a variant of Laver’s axiom which he proved inconsistent with the HOD Hypothesis when considered jointly with a smaller extendible cardinal (or a strongly compact cardinal, by Goldberg’s theorem)—namely, the existence of an elementary embedding $j:V_{\lambda +1}\to V_{\lambda +1}$ preserving $\Sigma_{2}$-truth in V. It is proved in ref. (9, Corollary 3.7) that Woodin’s hypothesis is equivalent to λ being ultraexacting.

## A Landscape of Large Cardinals

**Fig. 2.**
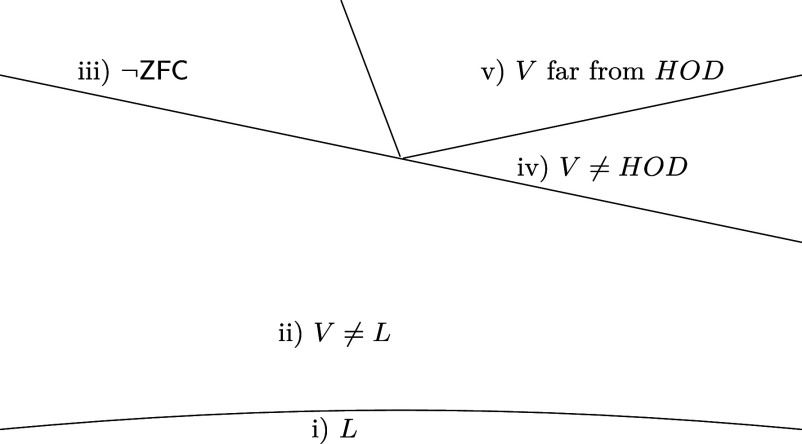
Proposed new view of dividing lines in the large-cardinal hierarchy.

The four inconsistency theorems suggest that the large-cardinal hierarchy might resemble Fig. 2 rather than Fig. 1. They may indicate that large cardinal axioms should be studied not only on the basis of their consistency strength, but also along a second axis: the degree to which they enforce failures of forms of the Axiom of Choice. Indeed, the results on ultraexacting cardinals and Schlutzenberg’s theorem show that there are equiconsistent cardinals in regions ii), iii), and iv) and according to Theorem 2, regions ii) and iv) overlap already at the level of exacting cardinals. It is proved in ref. (8, Theorem 6.5) that large cardinals in region iii) imply the consistency of those in v) which are known, and these are in turn obtained by combining the axioms in ii) and iv) (as in Theorem 3). The hypothesis of Theorem 3 is subtle, as it is proved in ref. (9, Theorem E) that I2 proves the consistency of ZFC together with the HOD Hypothesis and an exacting cardinal below a strongly compact cardinal (and more).

In the context of the future of set theory, large cardinals, and the foundations of mathematics, one nowadays speaks of a crossroads at which we find ourselves. The two new inconsistency theorems suggest that the crossroads diverge more than previously thought. If region v) is not vacuous and the HOD Conjecture fails, we find upon us a rich realm of infinity to explore and of mathematics to be developed beyond the realm of definable sets. If region v) is vacuous and the HOD Conjecture holds, we find upon us a divergence in the hierarchy of large cardinals, arguably with axioms ii) governing the orderly part of the set-theoretic universe, and axioms iv) governing the chaotic part thereof.

The authors believe that clarifying the relation between axioms ii), iii), iv), and v) is a crucial foundational issue in mathematics. We make the following series of predictions which, if confirmed or refuted, would aid in this endeavor.

Conjecture 1. *1. The theory* ZFC + *I0 proves the consistency of* ZFC *with an exacting cardinal above an extendible cardinal. 2. The theory* ZF + *“there is a rank-Berkeley cardinal” proves the consistency of* ZFC + *“there is an ultraexacting cardinal above an extendible cardinal.” 3. The theory* ZFC + *“there is an ultraexacting cardinal below an extendible cardinal” is consistent with the HOD Hypothesis*.

Question 1. *Which of the regions i)–v) dominate others in consistency strength?*

## Data Availability

There are no data underlying this work.
